# Supplementary material for: Cefotaxime Exposure-Caused Oxidative Stress, Intestinal Damage and Gut Microbial Disruption in Artemia sinica
Source: Microorganisms. 2024 Mar 28;12(4):675. doi: 10.3390/microorganisms12040675 (PMC11052325; doi:10.3390/microorganisms12040675)
Supplement: Supplementary file 1 [file microorganisms-12-00675-s001.zip › microorganisms-2908703-supplementary.pdf]

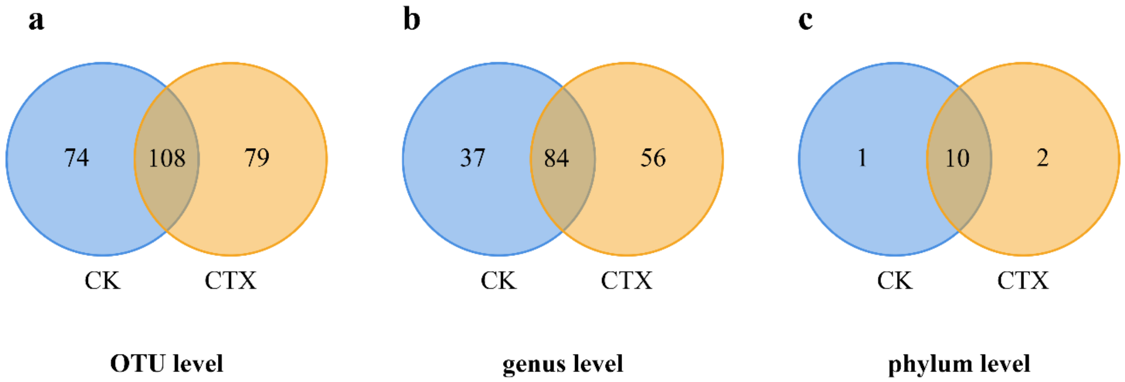

Figure S1 Venn plot representing the overlap of gut microbiome between the CK and CTX groups at (a) OUT level; (b) phylum level; and (c) genus level.

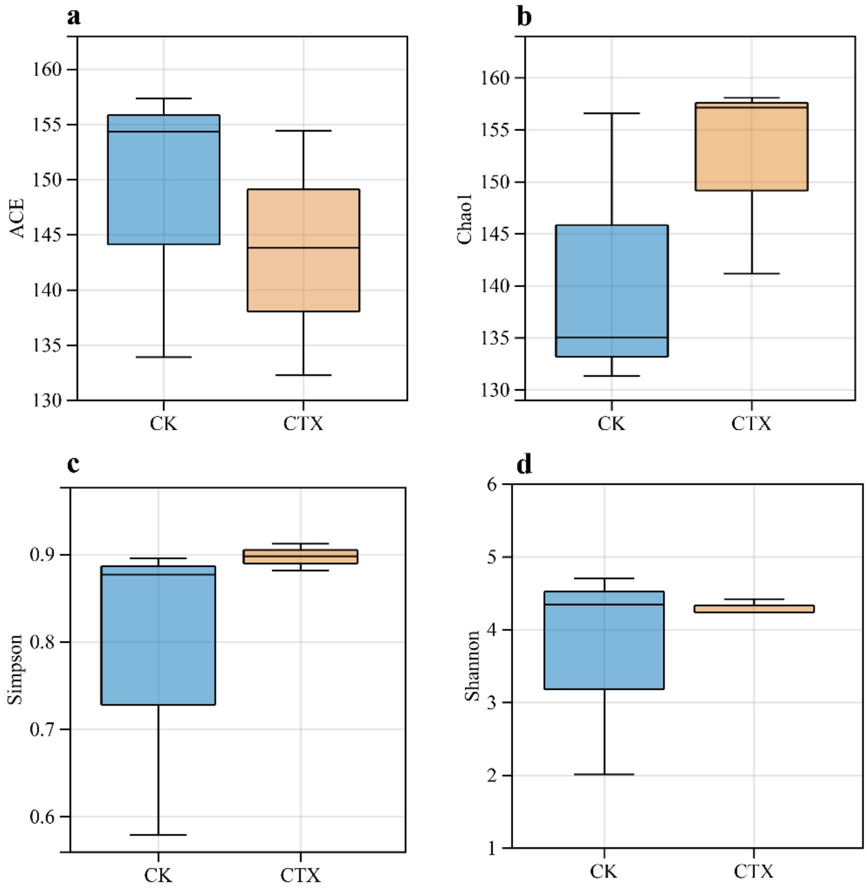

Figure S2 Alpha diversity of gut microbiota between the CK and CTX group reflected by (a) ACE; (b) Chao1; (c) Simpson; and (d) Shannon index.

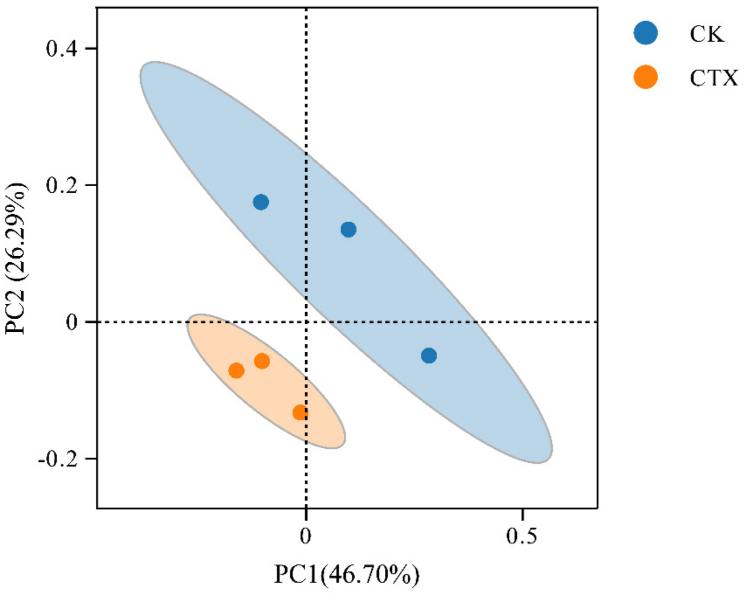

Figure S3 Principal component analysis (PCA) of samples in CK and CTX groups based on 16s rRNA gene sequencing. The plot showed the first two principal components.
